# Complementary and Alternative Therapies for Liver Diseases 2014

**DOI:** 10.1155/2015/476431

**Published:** 2015-07-09

**Authors:** Yong-Song Guan, Qing He, Mohammad Ahmad Al-Shatouri

**Affiliations:** ^1^Department of Oncology and Radiology, West China Hospital of Sichuan University, Chengdu, Sichuan 610041, China; ^2^Suez Canal Medical School, Suez Canal University, Ismailia 41522, Egypt

Liver diseases are now increasing, especially the chronic ones. The therapy of liver diseases is often challengeable because conditions of the liver are complicated in both structure and function. Different pathological liver conditions are associated with reactive oxygen species, viruses, toxins, inflammation and infection, and others. The resultant liver fibrosis and liver cirrhosis are a problem that every year thousands of people suffer from and many doctors are puzzled by. Liver cirrhosis, a potential cause of liver cancer, has long been considered irreversible or extremely difficult to reverse.

There has been a good deal of interest in complementary and alternative therapies for the treatment of various liver diseases. Patients generally appreciate the treatment of liver diseases with natural agents because of their diverse effects and mild side effects. One natural agent commonly has multiple therapeutic effects including antioxidant, anti-inflammatory, antiviral, and antitoxic properties. The selection of a plant or plants as a medicine needs sufficient data and definite proof of the efficacy in liver diseases.

This special issue provides original research, review, and meta-analysis about the recent information on complementary and alternative therapies for liver diseases involving* ex vivo*,* in vivo,* and clinical studies. A number of natural agents are deeply investigated and analyzed for the treatment of liver diseases on the basis of evidence in the aspects of cell signal pathways and other molecular mechanisms and clinical practice.

Readers of this journal are expected to find in this special issue recent developments not only in treatment but also in prevention of liver diseases by complementary and alternative methods. The readers might be interested in studies on Fuzheng-Huayu Formula against liver fibrosis and cirrhosis by developing healthy liver cells to replace the damaged ones. The cure of a cirrhotic liver is no longer like getting blood out of a stone.

*Yong-Song Guan* *Yong-Song Guan* *Qing He* *Qing He* *Mohammad Ahmad Al-Shatouri* *Mohammad Ahmad Al-Shatouri*

